# Population-based prevalence survey of follicular trachoma and trachomatous trichiasis in the Casamance region of Senegal

**DOI:** 10.1186/s12889-017-4605-0

**Published:** 2017-07-26

**Authors:** Emma M. Harding-Esch, Julbert Kadimpeul, Boubacar Sarr, Awa Sane, Souleymane Badji, Mass Laye, Ansumana Sillah, Sarah E. Burr, David MacLeod, Anna R. Last, Martin J. Holland, David C. Mabey, Robin L. Bailey

**Affiliations:** 1 Department of Clinical Research, London School of Hygiene & Tropical Medicine, London, UK; 2 Programme National de Lutte Contre la Cécité, Ministère de la Sante, Dakar, Sénégal; 3 National Eye Health Programme, Ministry of Health and Social Welfare, Kanifing, Gambia; 4 Disease Control and Elimination Theme, Medical Research Council Unit, The Gambia, Fajara, Banjul, Gambia; 5 Department of Infectious Disease Epidemiology, London School of Hygiene & Tropical Medicine, London, UK

**Keywords:** *Chlamydia trachomatis*, Active trachoma, Trachomatous trichiasis, Survey, Senegal, Control

## Abstract

**Background:**

Trachoma, caused by ocular infection with *Chlamydia trachomatis*, is the leading infectious cause of blindness worldwide. We conducted the first population-based trachoma prevalence survey in the Casamance region of Senegal to enable the Senegalese National Eye Care Programme (NECP) to plan its trachoma control activities. The World Health Organization (WHO) guidelines state that any individual with trachomatous trichiasis (TT) should be offered surgery, but that surgery should be prioritised where the prevalence is >0.1%, and that districts and communities with a trachomatous inflammation, follicular (TF) prevalence of ≥10% in 1–9 year-olds should receive mass antibiotic treatment annually for a minimum of three years, along with hygiene promotion and environmental improvement, before re-assessing the prevalence to determine whether treatment can be discontinued (when TF prevalence in 1–9 year-olds falls <5%).

**Methods:**

Local healthcare workers conducted a population-based household survey in four districts of the Bignona Department of Casamance region to estimate the prevalence of TF in 1–9 year-olds, and TT in ≥15 year-olds. Children’s facial cleanliness (ocular and/or nasal discharge, dirt on the face, flies on the face) was measured at time of examination. Risk factor questionnaires were completed at the household level.

**Results:**

Sixty communities participated with a total censused population of 5580 individuals. The cluster-, age- and sex-adjusted estimated prevalence of TF in 1–9 year-olds was 2.5% (95% Confidence Interval (CI) 1.8–3.6) (38/1425) at the regional level and <5% in all districts, although the upper 95%CI exceeded 5% in all but one district. The prevalence of TT in those aged ≥15 years was estimated to be 1.4% (95%CI 1.0–1.9) (40/2744) at the regional level and >1% in all districts.

**Conclusion:**

With a prevalence <5%, TF does not appear to be a significant public health problem in this region. However, TF monitoring and surveillance at sub-district level will be required to ensure that elimination targets are sustained and that TF does not re-emerge as a public health problem. TT surgery remains the priority for trachoma elimination efforts in the region, with an estimated 1819 TT surgeries to conduct.

## Background

Trachoma is the leading infectious cause of blindness worldwide [1]. Repeated ocular infections with the bacterium *Chlamydia trachomatis* can lead to subepithelial follicles (trachomatous inflammation, follicular (TF)) and/or inflammation (trachomatous inflammation, intense (TI)). TF and TI are referred to as active trachoma, which is usually found in children. After years of infection, trachomatous scarring (TS) can occur, resulting in the eyelid contracting and causing the eyelashes to turn inwards and scratch the cornea (trachomatous trichiasis, TT), leading to corneal opacity and blindness [2].

The World Health Assembly has set a target for the Global Elimination of Blinding Trachoma by the year 2020 (GET 2020) [3], to be achieved through implementation of the SAFE strategy for trachoma control: **S**urgery for TT, **A**ntibiotics to treat active disease, **F**ace washing and **E**nvironmental improvement. The latest World Health Organization (WHO) guidelines are that any individual with TT should be offered surgery, but that surgery should be prioritised where the prevalence is >0.1%. Elimination of TT as a public health problem is achieved when there are fewer than 2 cases of TT unknown to the health system per 1000 adults aged 15 years or above at the district level (or <1 case per 1000 total population) [4, 5]. Districts and communities with a TF prevalence of ≥10% in 1–9 year-olds should receive mass antibiotic treatment annually for a minimum of three years, before re-assessing the prevalence to determine whether treatment can be discontinued (when TF prevalence in 1–9 year-olds falls <5%) [4]. There are no recommendations for the use of facial cleanliness and environmental improvement indicators for the assessment of trachoma elimination, but these SAFE strategy components should be implemented at any TF prevalence level [4–6].

The dry, arid, Sahel belt of West Africa reports some of the highest prevalence rates for TF and TT [7]. In The Gambia, trachoma was the second leading cause of blindness in 1986 [8], but following formation of the National Eye Health Programme (NEHP) which implemented trachoma control activities, the prevalence of active trachoma in 0–14 year-olds decreased from 10.4% to 4.9% (a 54% reduction) by 1996 [9]. In 2006, TF prevalence in 1–9 year-olds remained ≥10% in two Gambian regions (12.3% and 10.0%) [10], and mass drug administration (MDA) with azithromycin was initiated in 11 rural districts. In 2011, results from the Partnership for the Rapid Elimination of Trachoma (PRET) showed that the overall TF prevalence in four districts was 2.8% (compared to 6.5% at baseline in 2008) [11]. The Gambia’s NEHP has subsequently reported national prevalences of TF ranging between 0.2 and 3.2%, and TT ranging between 0 and 1.7%, suggesting that The Gambia has reached the elimination target for TF but that further efforts are needed to reduce the burden of TT [12]. Similarly in Mali, the National Blindness Prevention Programme (PNLC)’s trachoma control programme formed in 1998 has helped reduce TF prevalence from a range of 23.1–46.7% in the 1990’s to below 10% in 84% of districts in 2013 [13]. Approximately 27,000 more TT surgeries are required to meet the elimination of trachoma as a public health problem target of fewer than two cases of TT unknown to the health system per 1000 persons aged ≥15 years [4, 5]. Trachoma prevalence data from Guinea Bissau are limited, but a population-based prevalence survey in the Bijagós Archipelago found 22.0% active trachoma in 1–9 year-olds, and prevalence of TT is reported to be 3.5% [14].

In Senegal, whose northern regions lie to the west of the Sahel area, the last national trachoma survey was conducted in 2000, and showed that 10.8% of children aged less than 10 years had active trachoma, 2.6% of women aged over 14 years had TT, and 1.4% of women aged more than 14 years had corneal opacity [15]. A study conducted in 2004 in the Nioro department (within Kaolack Region, which had a prevalence of 6.8% in the 2000 survey), had an active trachoma prevalence of 17.4% in children aged 2–5 years [16]. These results demonstrate that trachoma is a public health problem in Senegal, with heterogeneity throughout the country. The Senegalese national programme for trachoma control began in 2004, and mass treatment of districts commenced in 2005.

**Fig. 1:**
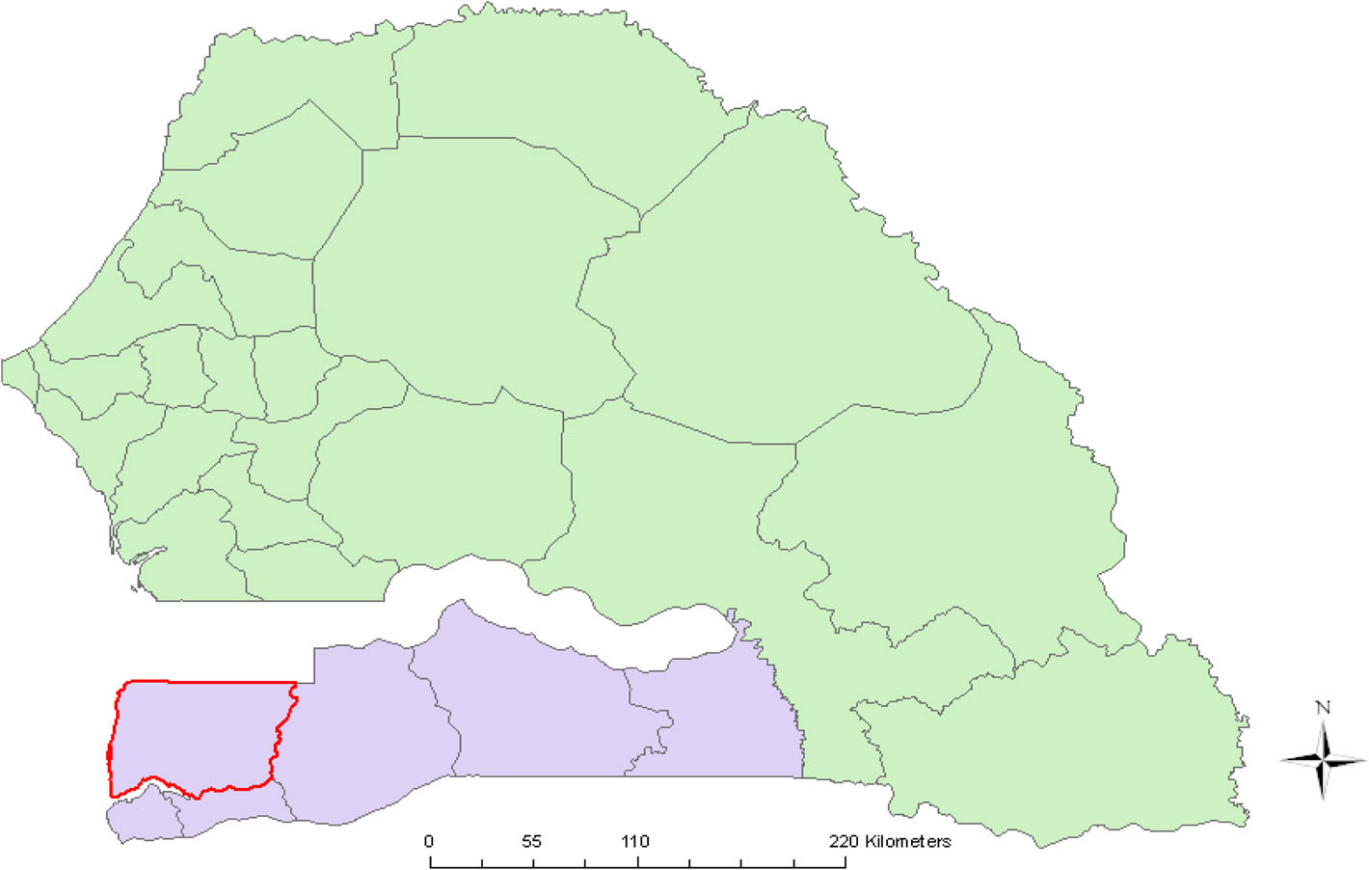
Map of Senegal. Casamance region shaded in purple. Bignona Department included in the survey highlighted in red. Source data: authors and open source information

The Casamance region of Senegal (Fig. 1), which underwent low-level conflict for approximately 20 years since the 1990’s, was not included in the national trachoma survey, and therefore the appropriate SAFE trachoma control efforts could not be targeted or implemented. A prevalence survey in the Casamance region was needed so that the Senegalese National Eye Care Programme (NECP) could plan its trachoma control activities (number of TT operations, requirements for mass antibiotic treatment, and whether hygiene and sanitation interventions were needed). Furthermore, capacity building was required in this region to enable the NECP to conduct its own trachoma prevalence surveys to assess control intervention needs and impact, and provide data to the WHO.

The aim of the study was to conduct a population-based TF and TT prevalence survey of the Bignona Department of the Casamance region of Senegal. The specific objectives were to:

1. Assess the prevalence of TF in children aged 1–9 years at the regional, district, commune and community levels.
2. Assess the prevalence of TT in adults aged ≥15 years at the regional, district, commune and community levels.

## Methods

### Training

To increase trachoma assessment capacity within the region, a total of 45 individuals (community members, village health workers and community nurses) from the Casamance region were selected by the Bignona cataract surgeon to be trained. Training took place between 7th–11th December 2009 (three classroom days and two days in the field) and encompassed trachoma grading based on the WHO simplified grading system [17] (practised every day using projected photographs with a focus on the clinical signs of active trachoma), survey methods, field practice of trachoma grading and data collection, and data recording. On the final day, the trainees were formally tested on their trachoma grading (grading projected photographs) and data recording skills (written exam). 40/45 trainees achieved the required trachoma grading agreement kappa score of ≥0.8 against the trainer, and were certified to perform trachoma grading for the survey. They formed four teams of ten trainees, five of whom did trachoma grading and five of whom filled in the forms. Fieldwork took place between January and May 2010.

### Sample size calculation

We used the WHO-recommended sample size calculation method for programme managers [6]. The WHO recommends that trachoma prevalence estimates should be collected at the district level, with population sizes between 100,000 and 250,000, as this is the normal administrative unit for SAFE implementation [4, 18]. A census (number of inhabitants only) had been conducted by the Casamance NECP team of all villages in the Bignona Department of Casamance (Fig. 1), comprising the districts of Diouloulou, Sindian, Tendouck and Tenghori, hereafter referred to as the regional level, totalling a population of 232,427. Thus the sample size calculation was based at the regional level, as this population size equates to that of a standard district according to WHO definitions.

Based on the census population size and the assumption, derived from previous work in the neighbouring country of The Gambia [19], that a third of the population was aged 1–9 years, it was estimated that there were 77,480 children in the region. Assuming an active trachoma prevalence of 20% [15, 20], using a precision estimate of ±5%, an alpha risk of 5%, and a design effect of 4, a sample size of 2732 children aged 1–9 years was calculated [6]. We set the cluster size to 50 children, thereby requiring 55 communities to be included in the survey. We included 60 communities, to ensure the sample size requirements were met. The total population size (232,427) was divided by the desired number of communities (60) to set the sampling interval (3874). A list of the censused communities and their cumulative population size was compiled. A random number between 1 and 3874 was selected and the community whose cumulative population equalled this number was the first community selected. The sampling interval was then added and communities selected from the cumulative population size list, until 60 communities were chosen.

Any community with a population above 1000 people was divided into two, using a logical demarcation (e.g. road), and one area was randomly selected for inclusion in the survey. Communities in the rural communes (the next administrative level after districts) of Sindian and Iles Karone, where higher trachoma prevalence was expected, were over-sampled by halving the sampling interval, to help identify trachoma hot-spots to ensure trachoma control efforts were appropriately implemented. Urban communes of Bignona, Tenghori Transgambienne, and Thionck-Essyl were under-sampled by doubling the sampling interval, based on the assumption that they would have lower prevalence and would be more difficult to sample and survey.

### Sampling selection

We followed standard population-based prevalence survey methodology [21]. We employed a two-stage cluster random sampling strategy, with probability of selection proportional to size. In the first stage, the 60 communities were randomly selected, resulting in 17, 18, 5 and 20 communities being selected in the districts of Diouloulou, Tenghori, Tendouck and Sindian, respectively (Table 1). In the second stage, we made a list of household heads with which to make a random selection of ten households in each selected community, with three reserve households in case any of the selected households could or would not participate.

**Table 1:** Prevalence of trachomatous inflammation, follicular (TF) and trachomatous trichiasis (TT)

		TF			TT		
Commune	No. communities	No. 1–9 year-olds with TF results	No. with TF (%)	Adjusted TF, % (95%CI)^a^	No. ≥15 year-olds with TT results	No. with TT (%)	Adjusted TT, % (95%CI)^a^
Region (Bignona Department of Casamance)	60	1425	38 (2.7)	2.5 (1.8–3.6)	2744	40 (1.5)	1.4 (1.0–1.9)
Sindian District	20	461	15 (3.3)	3.0 (1.5–5.9)	938	15 (1.6)	1.5 (0.8–2.7)
Suelle	3	63	1 (1.6)	1.7 (0.2–11.5)	123	4 (3.3)	3.2 (1.2–8.4)
Djibidione	2	46	1 (2.2)	2.0 (0.3–13.6)	64	0 (0.0)	0.0 (0.0–11.5)
Sindian^b^	6	136	4 (2.9)	3.0 (1.1–7.7)	297	4 (1.3)	1.3 (0.5–3.5)
Oulampane	5	147	8 (5.4)	5.4 (2.7–10.6)	281	6 (2.1)	2.1 (0.9–4.6)
Mangagoulack	2	30	0 (0.0)	0.0 (0.0–11.6)	79	1 (1.3)	1.3 (0.2–9.3)
Thionck-Essyl^c^	2	39	1 (2.6)	3.0 (0.4–19.8)	94	0 (0.0)	0.0 (0.0–13.5)
Tenghori District	18	389	6 (1.5)	1.8 (0.8–4.2)	812	10 (1.2)	1.1 (0.6–1.9)
Bignona^c^	5	100	2 (2.0)	2.0 (0.5–7.8)	265	2 (0.8)	0.7 (0.2–2.9)
Tenghori	3	61	1 (1.6)	1.6 (0.2–11.3)	98	2 (2.0)	1.8 (0.4–6.9)
Tenghori Transgambienne^c^	2	39	2 (5.1)	4.9 (1.2–18.7)	83	0 (0.0)	0.0 (0.0–17.3)
Ouonck	3	78	0 (0.0)	0.0 (0.0–4.6)	137	3 (2.2)	2.3 (0.7–6.9)
Coubalan	3	70	0 (0.0)	0.0 (0.0–5.1)	159	2 (1.3)	1.3 (0.3–5.3)
Niamone	2	41	1 (2.4)	2.7 (0.3–18.1)	70	1 (1.4)	1.7 (0.2–11.6)
Tendouck District	5	108	2 (1.9)	2.1 (0.4–9.7)	235	6 (2.6)	2.6 (1.7–4.0)
Balinghor	1	26	1 (3.8)	4.1 (0.5–26.9)	55	1 (1.8)	1.6 (0.2–11.4)
Karthiackk	2	32	1 (3.1)	3.3 (0.4–22.1)	79	2 (2.5)	2.7 (0.6–10.7)
Diegoune	2	50	0 (0.0)	0.0 (0.0–7.1)	101	3 (3.0)	2.9 (0.9–8.9)
Diouloulou District	17	467	15 (3.2)	3.1 (1.9–5.1)	759	9 (1.2)	1.2 (0.5–3.0)
Djinaky	4	97	3 (3.1)	2.3 (0.7–7.3)	190	2 (1.1)	0.9 (0.2–3.4)
Kafountine	4	106	4 (3.8)	3.8 (1.4–9.8)	183	1 (0.5)	0.5 (0.1–3.7)
Iles Karone^b^	3	47	2 (4.3)	4.6 (1.1–17.3)	80	4 (5.0)	4.9 (1.8–12.5)
Diouloulou	6	217	6 (2.8)	2.8 (1.3–6.1)	306	2 (0.7)	0.7 (0.2–2.7)

^a^Prevalences are adjusted for non-response by age and sex. Region and district estimates are additionally weighted by commune size. 95% Confidence Intervals (95%CIs) are logit-transformed confidence limits. Where prevalence is zero, one-sided 97.5% Clopper-Pearson exact CIs are presented

^b^Rural commune (communities over-sampled by halving the sampling interval)

^c^Urban commune (communities under-sampled by doubling the sampling interval)

The survey teams then enumerated all household members who had slept in the household the night before (the de facto population) for the randomly selected households in the randomly selected communities. Risk factor questionnaires, including questions on sex of the household head, ethnic group, occupation, education level, access to a latrine, type of water source and time to fetch water (return journey), were completed. The questions were based on those from questionnaires previously used in The Gambia [22]; responses were not confirmed by observational data.

Attempts were made to examine the eyes of all household members recorded during the census for trachoma clinical signs according to the WHO simplified grading system [17]: trachomatous inflammation, follicular (TF), trachomatous inflammation, intense (TI), trachomatous scarring (TS), trachomatous trichiasis (TT) and corneal opacity (CO). Dirt on the face, nasal and ocular discharge, and flies on the face at the time of examination (measures of facial cleanliness) were recorded. The examination team returned to households where censused individuals were not present for the first examination visit, in order to increase screening rates.

### Statistical analyses

Data were double entered by different data entry clerks into a Microsoft Access database (v. 2007). Data cleaning and analyses were conducted in Stata (v12, STATA Corp., College Station, TX, USA). Discrepancies between the databases were resolved by a third individual by referring to the paper forms.

TF and TT prevalences and their 95% Confidence Intervals (CIs) were estimated for children aged 1–9 years and for individuals aged ≥15 years, respectively, using Stata’s “svy: proportions” commands. These estimates were weighted to allow for under/over sampling of communes, using a weight calculated by dividing the proportion of the region that would be expected in the sample (if each commune was sampled proportional to size) by the proportion observed in the sample. The data were also weighted by age and gender to allow for differences in non-response across age groups and genders. Age was weighted using three-year age bands for 1–9 year-olds, and five-year age bands for ≥15 year-olds. Where prevalence was 0%, the CIs were calculated using the Clopper-Pearson exact method.

Prevalence is reported at region and district levels for healthcare management purposes [4], and at commune and community levels for epidemiological interest purposes. Characteristics from the risk factor questionnaire are reported for the censused population, children (aged 1–9 years) examined, and adults (aged ≥15 years) examined, but formal risk factor analyses were not conducted due to insufficient statistical power resulting from low trachoma prevalence.

The spatial distribution of TF and TT prevalence is presented using ArcMap v9.2 (Environmental Systems Research Institute, Inc. Redlands, CA, USA). Community GPS coordinates were collected in the field. Open source maps (www.openstreetmap.org) were obtained from CloudMade.org.

## Results

### Study participation

Of the 60 randomly selected communities, all consented to participate. Ten randomly selected households per community were included, except in one community where only nine households were approached. A total of 5580 individuals were censused, of which 1554 (27.8%) were aged 1–9 years (supporting the TF sample size calculation assumption) and 3121 (55.9%) were aged ≥15 years (Table 2). A total of 1432 children aged 1–9 years were examined, and 2750 adults aged ≥15 years. Individual participation in the study was high, at 92.1% for 1–9 year-olds and 88.1% for those aged ≥15 years (Table 2). 90.4% of censused females were examined for TT compared to 85.4% of males (chi-squared *p* = 0.287) (Table 2).

**Table 2:** Age and sex distribution of participants censused and examined

Age (years)	Number censused^a^	Number (%) examined	Number (%) not examined		
			Absent	Refused	Other reason/no reason provided
Males					
1–9	813	742 (91.3)^b^	70 (8.6)	0 (0.0)	1 (0.1)
≥15	1421	1214 (85.4)	196 (13.8)	5 (0.4)	6 (0.4)
Females					
1–9	741	690 (93.1)^b^	47 (6.3)	4 (0.5)	0 (0.0)
≥15	1700	1536 (90.4)	161 (9.5)	3 (0.2)	0 (0.0)
Total	4675	4182 (89.5)	474 (10.1)	12 (0.3)	7 (0.1)

^a^Age missing for 48 individuals

^b^Could not evert eyelid in seven examined children aged 1–9 years (3 males, 4 females)

### Overview of study population

Children’s faces were relatively clean, with <6% recorded as having dirt on their faces, ocular discharge or flies on their faces at the time of examination (Table 3). However, 14.7% were reported as having nasal discharge. The majority (82.2%) of household heads were male, with Diola the dominant ethnic group (82.4%), and agriculture was the main occupation (66.3%). Over half of adults had attended school (54.2%), the majority to primary level only (59.0%). Most households (88.4%) had access to a latrine, with over half of these (52.4%) being privately owned. The predominant water source was an uncovered well outside of the household (51.0%), with most adults (90.4%) reporting it took <30 min to fetch water.

**Table 3:** Population characteristics

Characteristic	No. (%)^a^
Total censused population	5580
Sex	
Male	2726 (48.9)
Female	2854 (51.1)
Age (years)	
1–9	1554 (27.8)
≥15	3121 (55.9)
Examined children (1–9 years) characteristics	1432
Dirt on the face	77 (5.4)
Ocular discharge	35 (2.4)
Nasal discharge	210 (14.7)
Flies on face at time of examination	5 (0.3)
Examined adult (≥15 years) characteristics	3121
Household head characteristics	
Sex	
Male	2564 (82.2)
Female	557 (17.8)
Ethnicity	
Diola	2573 (82.4)
Peulh	98 (3.1)
Mandingue	164 (5.3)
Other	286 (9.2)
Occupation	
Agriculture	2068 (66.3)
Trader	140 (4.5)
Teacher	109 (3.5)
No activity	123 (3.9)
Other	681 (21.8)
Whether attended formal education	
Yes	1690 (54.2)
No	1429 (45.8)
Highest education level reached	
Primary	997 (59.0)
Secondary	621 (36.7)
University	72 (4.3)
Household characteristics	
Access to a latrine (shared or private)	
Yes	2754 (88.4)
No	360 (11.6)
Latrine ownership	
Shared	1312 (47.6)
Private	1442 (52.4)
Principal water source	
Well inside household	925 (29.7)
Tap inside household	444 (14.2)
Uncovered well outside household	1590 (51.0)
Outside covered well with pump	67 (2.1)
Outside tap	51 (1.6)
Other	42 (1.3)
Habitual time to fetch water^b^	
Less than time to cook rice	2811 (90.4)
Same as the time to cook rice	190 (6.1)
More than the time to cook rice	110 (3.5)

^a^Where data were missing, percentages are based on denominators for which data are available

^b^Time was contextualised as the time to cook rice (approximately 30 min)

### Prevalence of clinical signs

The prevalences presented for TF and TT are weighted for the cluster size relative to the total community population size as per the census provided by the Casamance region NECP team, and age- and sex-adjusted against those censused, but not examined, in our study.

### TF in 1–9 year-olds

One thousand four hundred thirty-two children aged 1–9 years were examined for clinical signs of trachoma, with TF results available for 1425 (Table 1). At the regional level, TF was found in 38 (2.5%, 95%CI 1.8–3.6) children aged 1–9 years. At the district level, the prevalence of TF was <5% in all districts, although the upper 95%CI exceeded 5% in all but the district of Tenghori.

At the commune level, none of the 19 communes had a prevalence ≥10%, and only one (Oulampane) had a TF prevalence ≥5%. The rural communes of Sindian and Iles Karone, in which communities were over-sampled due to expected higher trachoma prevalence, had the 8th and 3rd highest TF prevalences, at 3.0% (95%CI 1.1–7.7) and 4.6% (95%CI 1.1–17.3), respectively. The urban communes (Bignona, Tenghori Transgambienne and Thionck-Essyl) in which communities were under-sampled due to expected lower trachoma prevalence and difficult survey logistics, had the 2nd, 7th and 12th highest TF prevalences. The highest TF prevalence commune was Oulampane (5.4%, 95%CI 2.7–10.6) in Sindian district.

**Fig. 2:**
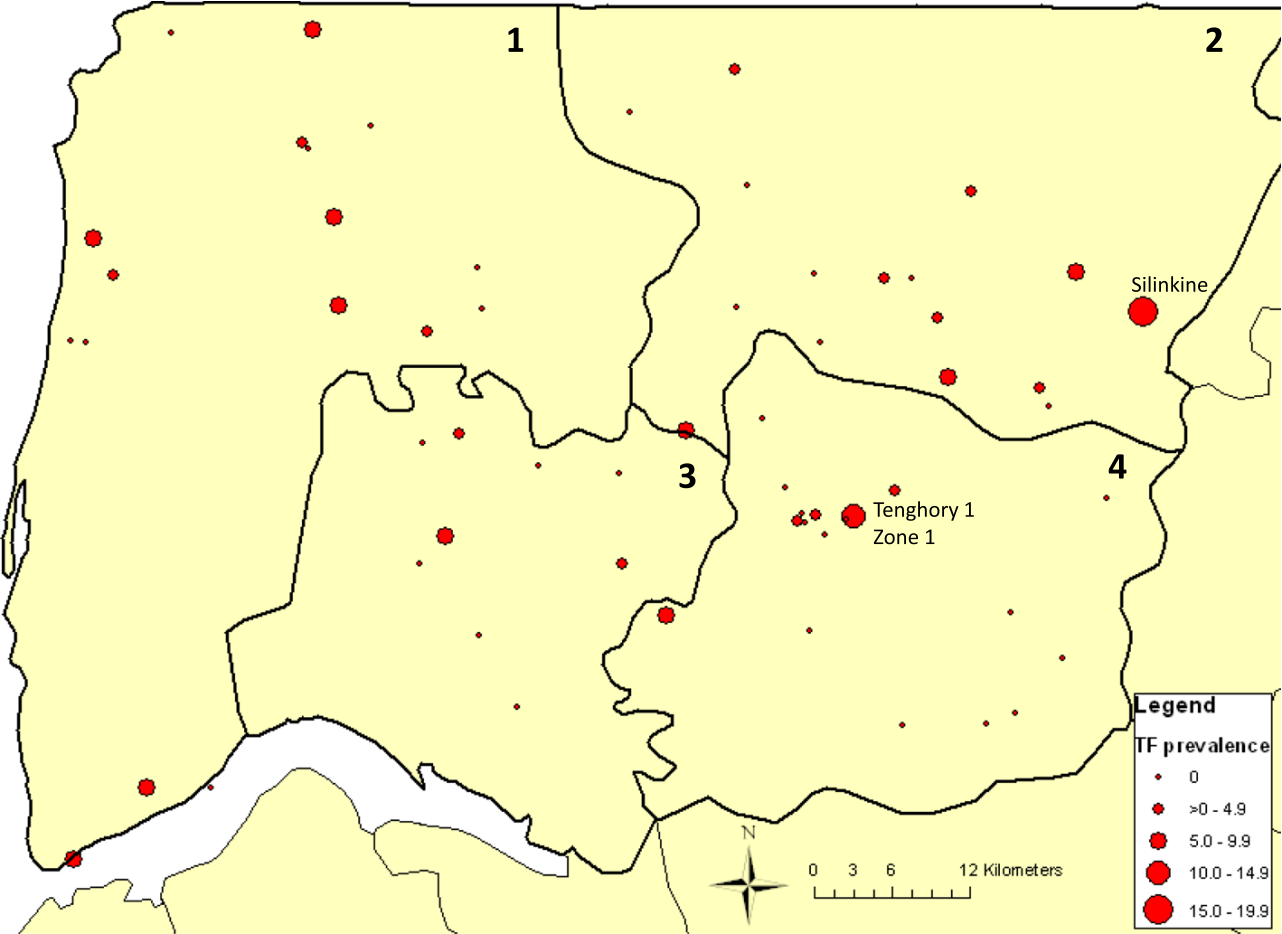
Point prevalence of trachomatous inflammation, follicular (TF) at community level in 1–9 year-olds. Districts: 1 = Dioloulou; 2 = Sindian; 3 = Tendouck; 4 = Tenghori. Source data: authors and open source information

At the community level, only two of the 60 communities had a TF prevalence ≥10% (Tenghory 1 zone 1 in the commune of Tenghori Transgambienne (10.3%, 95%CI 1.5–45.8), and Silinkine in the commune of Oulampane (15.2%, 95%CI 1.5–67.7)) (Fig. 2), and 10 had a prevalence between 5% and 10%. Thirty-four communities had a prevalence of 0%.

### Trachomatous trichiasis (TT) in ≥15 year-olds

Two thousand seven hundred fifty adults aged ≥15 years were examined, with TT results available for 2744 (Table 1). At the regional level, a total of 40 TT cases was found (1.4%, 95% CI 1.0–1.9) in individuals aged ≥15 years. Prevalence by sex was 0.8% (95%CI 0.4–1.6; 10/1213) in males and 1.8% (95%CI 1.3–2.4; 30/1531) in females. After adjusting for age, there was evidence (*p* = 0.024) that the odds of TT were higher among females than males, with females estimated to have 2.3 times the odds of TT compared to males (95%CI 1.1–4.6). At the district level, the prevalence of TT was >1% in all districts and the lower 95%CI exceeded 0.1% in all districts.

**Fig. 3:**
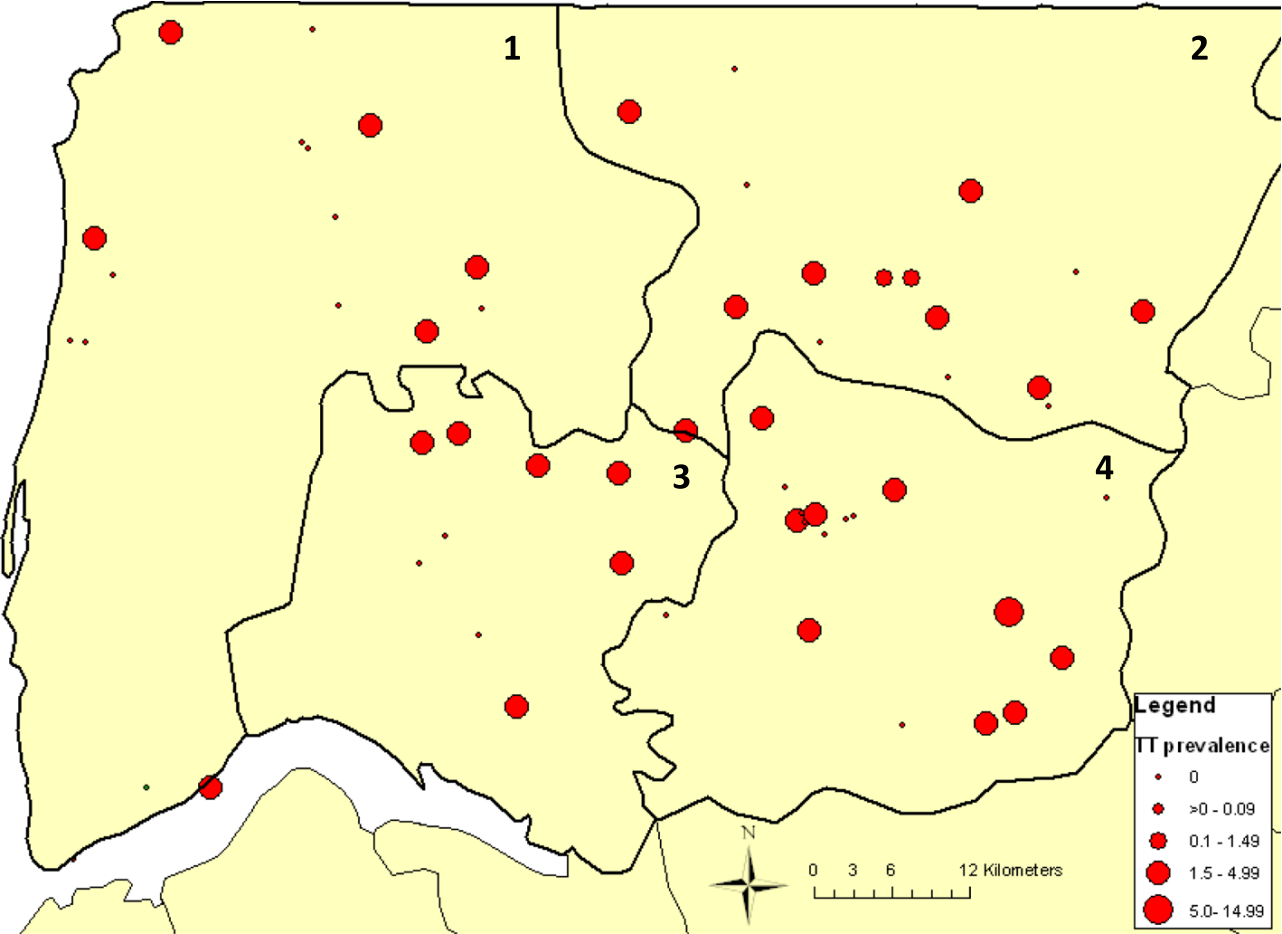
Point prevalence of trachomatous trichiasis (TT) at community level in ≥15 year-olds. Districts: 1 = Dioloulou; 2 = Sindian; 3 = Tendouck; 4 = Tenghori. Source data: authors and open source information

At the commune level, three communes had a prevalence of 0%, seven a prevalence of between 0.1% and 1.5%, and the remaining nine had a prevalence ≥1.5% (range 1.6–4.9%) (Table 1). The Iles Karone commune, in which communities were over-sampled due to expected higher trachoma prevalence, had the highest TT prevalence of all communes (4.9%, 95%CI 1.8–12.5), whereas Sindian’s TT prevalence was in-line with other communes at 1.3% (95%CI 0.5–354). Two of the three communes with 0% TT were from the communes that had been under-sampled due to expected lower trachoma prevalence (Tenghori Transgambienne and Thionck-Essyl), and Bignona had a prevalence of 0.7% (95%CI 0.2–2.9). At the community level, 28 of the 60 communities had a TT prevalence of 0% and the remaining 32 had a prevalence ≥1% (range 1.2–14.8%) (Fig. 3).

### Other trachoma grades (TI, TS, CO)

The crude prevalence of trachomatous inflammation, intense (TI) in all examined individuals was 0.8%, 95%CI 0.6–1.1 (41/4979; the eyelid could not be everted for 26 individuals), and 0.5%, 95%CI 0.2–1.0 (7/1425) in children aged 1–9 years (seven eyelids could not be everted). The prevalence of trachomatous scarring (TS) in all individuals was 1.6%, 95%CI 1.2–2.0 (81/4979), and 2.5%, 95%CI 2.0–3.2 in those aged ≥15 years (69/2740; eyelid could not be everted for 10 individuals, the reason for which was not recorded). Of those aged ≥15 years with data available, 15.4% (6/39) of those with TT had TS, and 2.3% (63/2701) of those without TT had TS (chi-squared *p* < 0.001). The overall prevalence of corneal opacity (CO) was 0.9%, 95%CI 0.6–1.1 (43/4993), and 1.5%, 95%CI 1.1–2.0 (41/2744) in those aged ≥15 years.

## Discussion

This is the first trachoma survey to be conducted in the Bignona Department of Casamance in the Casamance region of Senegal. The estimated prevalence of TF in 1–9 year olds was 2.5% (95% CI 1.8–3.6) and the prevalence of TT in adults over the age of 14 years was 1.4% (95% CI 1.0–1.9). Both the TF and TT prevalences are lower than those reported in Senegal’s national trachoma survey conducted in 2000 (10.8% active trachoma in children <10 years, and 2.6% TT in women aged >14 years) [15]. The prevalence of TT in women in our study was 1.8% (95%CI 1.3–2.4).

The WHO determines that trachoma elimination has been achieved in a country if the TF prevalence in 1–9 year olds is <5% (precision of 4%, with a confidence interval of 2%) and the prevalence of TT cases unknown to the health system is <0.2% (2 cases per 1000 people aged ≥15 years) [4, 5]. In order to verify elimination, countries are required to meet WHO criteria for elimination and to demonstrate that this is sustainable for at least three years following cessation of programmatic interventions.

The WHO implementation units for trachoma elimination purposes are defined as districts (population between 100,000–250,000 individuals), which are the normal administrative unit for health care management, sub-districts (groupings of at least three villages permitting sub-unit stratification of a district) and villages (population between 8000 and 20,000 individuals) [5]. As the Casamance district population sizes were 66,135 (Sindian), 91,322 (Tenghori), 18,033 (Tendouck) and 56,937 (Diouloulou), we interpreted prevalence data at the Bignona Department of Casamance regional level, as opposed to the district level, because its population size of 232,427 equates to that of a WHO standard district level administrative unit [4, 18]. The TF prevalence at the regional level in 1–9 year olds was lower than the 10% WHO threshold, indicating that community MDA is not required (although facial cleanliness and environmental improvement efforts may continue), and was lower than the 5% threshold, indicating that TF is not a significant public health problem in this region or its districts [4, 6]. This is supported by the low TI prevalence of 0.5% in 1–9 year-olds, especially as TI is less specific as a clinical sign of trachoma than TF is [23].

Despite the encouraging TF results at the regional level, of the 60 communities surveyed in the Bignona Department of Casamance, two had a TF prevalence ≥10%, indicating they may require annual MDA for three years in addition to interventions promoting facial cleanliness and environmental improvement as proposed by the SAFE strategy [6]. Further investigation of these two, and their surrounding, communities is warranted to determine whether or not this higher TF prevalence is limited to these two communities [4]. However, as the TF prevalence was <5% at both regional and district levels, these communities are likely “the tail of the decline” [4]. The 10 communities with 5–10% TF prevalence are recommended to receive facial cleanliness and environmental improvement interventions, and in the remaining 48 communities, TF control is not currently a priority [4]. In this setting, sub-district community level decisions regarding MDA are required to ensure that trachoma elimination goals are achieved [4].

Furthermore, continued monitoring and surveillance are required in this setting, as re-emergence of ocular *C. trachomatis* infection and active trachoma has been documented in The Gambia following cessation of MDA [24]. Additionally, the Casamance region borders Guinea Bissau to the south, where TF prevalence in 1–9 year olds was estimated to be ≥20% in some districts. Though Guinea Bissau has now completed three rounds of MDA in most regions and is currently conducting impact surveys, this remains a potential concern. As a result of this risk of re-emergence, Neglected Tropical Disease (NTD) Programmes should regularly monitor districts with previous disease to ensure that it does not re-emerge as a significant public health problem [5]. National and regional surveillance systems should be employed to achieve this and should consider both active and passive surveillance for TF and TT case-finding [5, 25].

The low TF prevalence in the Casamance region was unexpected since no specific trachoma control efforts have been implemented there, unlike in other regions of Senegal or the neighbouring countries of The Gambia and Mali [12, 13]. The natural disappearance of trachoma without trachoma-specific intervention has been described elsewhere in Nepal and in one village in The Gambia [26, 27]. These findings are thought to be associated with the alleviation of poverty and improvements in sanitation, water supply, education and health care [27]. Senegal’s Human Development Index (HDI) improved from 0.367 in 1990, to 0.380 in 2000, and to 0.456 in 2010. This pattern is similar to that for The Gambia, whose HDI value increased from 0.330 in 1990, to 0.384 in 2000, to 0.441 in 2010 [28]. Thus, it is possible that a TF prevalence decrease due to secular trend, i.e. in the absence of trachoma control programmes, may have occurred. This explanation is supported by the high TT prevalence, which may represent the clinical sequelae from a previously higher burden of TF.

A component part of the HDI measure is educational attainment, and low household head educational attainment has been associated with TF in some countries such as Tanzania [19], but not in The Gambia [19, 22]. The household head education levels in our study were similar to those recorded in The Gambia [22], with 54.2% having attended school and two-thirds finishing with primary-level education. Thus, improvements in educational attainment are required in this region before achieving the Millennium Development Goal of universal primary education [29].

The risk factor questionnaire enquired about ethnicity, as the Casamance region population is distinct from other Senegalese regions, with the predominant ethnic group being Diola (82.4% in our study). Nationally, 44% of the population is of Wolof ethnicity, compared with 5% of Diola ethnicity. There are no reported ethnic group associations with trachoma in Senegal, and as the prevalences of TF and TT were too low to conduct formal risk factor analyses in our study, it was not possible for us to explore any associations in our population. Further investigations to assess whether ethnicity is related to health outcomes could nonetheless be warranted, as ethnicity has been associated with health outcomes (such as child mortality [30]) in this region.

The environmental risk factor data provide evidence of good water and sanitation levels in the Casamance region, and may also help explain the low prevalence of TF we observed. The proportion of children with unclean faces (dirt on the face (5.4%), ocular discharge (2.4%), nasal discharge (14.7%), flies on the face at the time of examination (0.3%)) was less than that found by Faye et al. in a Trachoma Rapid Assessment conducted in Senegal’s Kaolack region, where 12.5% of children aged 2–5 years had dirty faces and 4.6% had flies on their faces [16]. Latrine access was also higher in our study at 88.4% (compared to 60.9%), and 90.4% of respondents in our study reported that the time to fetch water was <30 min, whereas the water source was <25 m away for only 21.3% of those in Faye et al.’s study [16]. Environmental factors such as latrine and water access and use have been associated with active trachoma [31]. Latrines likely lead to reduced fly-eye contact, ultimately hindering transmission [32] – the high levels of latrine access in our study may explain the low proportion of children with flies observed on their faces. Access to water has been associated with reduced active trachoma, so long as it is used for hygiene purposes [33]. However, as our questionnaire was designed to be simple and quick in order to increase participation and completion, whilst enabling us to assess basic environmental conditions, we did not support the responses with observations of latrine use or measurements of water use practices, meaning we are unable to verify the responses given.

In striking contrast to the low TF prevalence, TT prevalence was high and far exceeded the WHO target for elimination as a public health problem of 0.2% (2 cases per 1000 adults aged ≥15 years) at both the regional and district levels [4, 5]. We found TT to be more prevalent in females than in males (1.8% versus 0.8%), with females having 2.3 times the odds of having TT than males. This association, which has been reported by others and reviewed with a meta-analysis showing a 1.8-fold higher risk of TT in females than males globally [34], is probably because women are the predominant caregivers and are in closer contact with children [35, 36]. However, other explanations such as females suffering higher loads of infection, being more prone to persistent infection, and/or being more biologically susceptible to consequences of *C. trachomatis* infection, may also play a role [35].

The natural history of trachoma posits that TS leads to TT [2], and there are several non-trachomatous causes of trichiasis, including inflammation, genetic defects and traumas [5, 37]. Although TT was significantly associated with having TS, with only 6/39 (15.4%) of individuals with TT also having TS recorded, our reported TT prevalence could be an over-estimate and could in fact be as low as 0.2% (15.4% of 1.4%). However, there is poor inter- and intra-observer agreement of TS grading, and consequently it is has not been included in the Global Trachoma Mapping Project methodology [38]. As the graders who conducted this survey were trained and assessed with a focus on TF and TT identification, we have decided to attribute the trichiasis cases recorded to a trachomatous aetiology. However, further work in this region to investigate whether the trichiasis is in fact due to trachoma, or other causes, is warranted. Notably, recent trachoma surveillance recommendations are that “the presence of scar, or the inability to evert the lid due to lid tightness, should be taken to indicate that the trichiasis is TT” in order to avoid misdiagnosis of TT [5].

The WHO recommends that all individuals with TT should be offered surgery, and that surgery should be prioritised where the prevalence is ≥0.1%. Based on the regional population size of 232,427, our census data indicating that 55.9% of the population is aged ≥15 years, and a TT prevalence of 1.4%, there are an estimated 1819 TT surgeries to conduct. Guidelines for TT management are provided by the WHO [1, 25]. One strategy is active TT case-hunting and surgery camps, as done in The Gambia, with surgeries recorded in a central, electronic TT registry to enable consistent data collection from across the region and ensuring appropriate follow-up care of all cases identified [12].

Limitations of the study are that there was not sufficient statistical power to conduct a risk factor analysis for TF due to the low prevalence, and that the sample size target of 2732 children aged 1–9 years was not achieved, with only 1432 children examined. Strengths of the study include providing the first data on TF and TT prevalence in this region, obtaining environmental data within which to contextualise the prevalence data, and capacity building within the region by having successfully trained 40 individuals from the region through a mixture of workshops and field exercises.

## Conclusions

The prevalence of TF in 1–9 year olds in the region and its districts was below the WHO 5% threshold for elimination, indicating that trachoma control for TF is not a priority in this setting. However, adequate monitoring and surveillance of TF at sub-district level will be required to ensure that elimination targets are sustained and that TF does not re-emerge as a public health problem. TT surgery remains the priority for trachoma elimination efforts in the region. The prevalence of TT far exceeds the 0.2% WHO threshold in adults aged ≥15 years for elimination of trachoma as a public health problem, with surgery needing to be prioritised urgently to meet the WHO elimination targets.

## Abbreviations

CI: Confidence interval
CNRS: National Centre for Scientific Research
GTMP: Global Trachoma Mapping Project
HDI: Human Development Index
MDA: Mass drug administration
NECP: National Eye Care Programme
NEHP: National Eye Health Programme
NTD: Neglected Tropical Disease
PNLC: National Blindness Prevention Programme
PRET: Partnership for the Rapid Elimination of Trachoma
SAFE: Surgery, Antibiotics, Facial cleanliness, Environmental Improvement
TF: Trachomatous inflammation, follicular
TI: Trachomatous inflammation, intense
TS: Trachomatous scarring
TT: Trachomatous trichiasis
WHO: World Health Organization

## References

[1] WHO (2014). WHO alliance for the global elimination of blinding trachoma by the year 2020. Progress report on elimination of trachoma, 2013. Wkly Epidemiol Rec.

[2] Mabey DC, Solomon AW, Foster A (2003). Trachoma. Lancet (London, England).

[3] WHO (1998). World health assembly. Global elimination of blinding trachoma. 51st world health assembly, Geneva, 16 may 1998, resolution WHA51.11.

[4] WHO. Report of the third global scientific meeting on trachoma. http://www.who.int/blindness/publications/WORLDHEALTHORGANIZATIONGSMmtgreportFINALVERSION.pdf?ua=1: Baltimore, 19-20th July 2010.

[5] WHO (2015). Technical consultation on trachoma surveillance, September 11–12, 2014. Technical advisory group on neglected tropical diseases.

[6] WHO (2006). Trachoma control - a guide for programme managers.

[7] Smith JL, Flueckiger RM, Hooper PJ, Polack S, Cromwell EA, Palmer SL (2013). The geographical distribution and burden of trachoma in Africa. PLoS Negl Trop Dis.

[8] Faal H, Minassian D, Sowa S, Foster A (1989). National survey of blindness and low vision in the Gambia: results. Br J Ophthalmol.

[9] Dolin PJ, Faal H, Johnson GJ, Ajewole J, Mohamed AA, Lee PS (1998). Trachoma in the Gambia. Br J Ophthalmol.

[10] Harding-Esch EM, Edwards T, Sillah A, Sarr I, Roberts CH, Snell P (2009). Active trachoma and ocular Chlamydia trachomatis infection in two Gambian regions: on course for elimination by 2020?. PLoS Negl Trop Dis.

[11] Harding-Esch EM, Sillah A, Edwards T, Burr SE, Hart JD, Joof H (2013). Mass treatment with azithromycin for trachoma: when is one round enough? Results from the PRET trial in the Gambia. PLoS Negl Trop Dis.

[12] Burr SE, Sillah A, Sanou AS, Wadagni AC, Hart J, Harding-Esch EM (2016). Cross-sectional surveys of the prevalence of follicular trachoma and Trichiasis in the Gambia: has elimination been reached?. PLoS Negl Trop Dis.

[13] PNLC HKI, CC. (2013). Mali: achieving success along the path to trachoma elimination. Community eye health.

[14] Thompson K, Hutchins H, Baio A, Cassama E, Nabicassa M, Bailey R (2015). Health beliefs and perceptions of trachoma in communities on the Bijagos archipelago of Guinea Bissau. Ophthalmic Epidemiol.

[15] Saal MB, Schemann JF, Saar B, Faye M, Momo G, Mariotti S (2003). Trachoma in Senegal: results of a national survey. Medecine tropicale.

[16] Faye M, Kuper H, Dineen B, Bailey R (2006). Rapid assessment for prioritisation of trachoma control at community level in one district of the Kaolack region, Senegal. Trans R Soc Trop Med Hyg.

[17] Thylefors B, Dawson CR, Jones BR, West SK, Taylor HR (1987). A simple system for the assessment of trachoma and its complications. Bull World Health Organ.

[18] Solomon AW, Kurylo E (2014). The global trachoma mapping project. Community eye health / International Centre for Eye Health.

[19] Harding-Esch EM, Edwards T, Mkocha H, Munoz B, Holland MJ, Burr SE (2010). Trachoma prevalence and associated risk factors in the Gambia and Tanzania: baseline results of a cluster randomised controlled trial. PLoS Negl Trop Dis.

[20] Ngondi J, Reacher M, Matthews F, Brayne C, Emerson P (2009). Trachoma survey methods: a literature review. Bull World Health Organ.

[21] Last AR, Burr SE, Weiss HA, Harding-Esch EM, Cassama E, Nabicassa M, et al. Risk factors for active trachoma and ocular Chlamydia trachomatis infection in treatment-naive trachoma-hyperendemic communities of the Bijagos archipelago, Guinea Bissau. PLoS Negl Trop Dis. 2014;8(6):e2900. doi:10.1371/journal.pntd.0002900. 10.1371/journal.pntd.0002900 PMC4072588 24967629

[22] Harding-Esch EM, Edwards T, Sillah A, Sarr-Sissoho I, Aryee EA, Snell P (2008). Risk factors for active trachoma in the Gambia. Trans R Soc Trop Med Hyg.

[23] Solomon AW, Peeling RW, Foster A, Mabey DC (2004). Diagnosis and assessment of trachoma. Clin Microbiol Rev.

[24] Burton MJ, Holland MJ, Makalo P, Aryee EA, Alexander ND, Sillah A (2005). Re-emergence of Chlamydia trachomatis infection after mass antibiotic treatment of a trachoma-endemic Gambian community: a longitudinal study. Lancet (London, England).

[25] WHO. Report on the Meeting on post-endemic Surveillance for Blinding Trachoma. http://www.who.int/blindness/publications/MEETINGONTRACHOMASURVEILLANCESYSTEM2008FINALVERSION.pdf Geneva, 4-5th November; 2008.

[26] Jha H, Chaudary JS, Bhatta R, Miao Y, Osaki-Holm S, Gaynor B (2002). Disappearance of trachoma from western Nepal. Clinical infectious diseases.

[27] Dolin PJ, Faal H, Johnson GJ, Minassian D, Sowa S, Day S (1997). Reduction of trachoma in a sub-Saharan village in absence of a disease control programme. Lancet (London, England).

[28] UNDP. Human Development Report 2015: Work for Human Development. http://hdr.undp.org/en/composite/trends United Nations Development Programme; Accessed 1 Mar 2017.

[29] UNDP (2015). The millennium development goals report.

[30] Brockerhoff M, Hewett P (2000). Inequality of child mortality among ethnic groups in sub-Saharan Africa. Bull World Health Organ.

[31] Emerson PM, Cairncross S, Bailey RL, Mabey DC (2000). Review of the evidence base for the ‘F’ and ‘E’ components of the SAFE strategy for trachoma control. Tropical medicine & international health.

[32] Emerson PM, Lindsay SW, Alexander N, Bah M, Dibba SM, Faal HB (2004). Role of flies and provision of latrines in trachoma control: cluster-randomised controlled trial. Lancet (London, England).

[33] Stocks ME, Ogden S, Haddad D, Addiss DG, McGuire C, Freeman MC (2014). Effect of water, sanitation, and hygiene on the prevention of trachoma: a systematic review and meta-analysis. PLoS Med.

[34] Cromwell EA, Courtright P, King JD, Rotondo LA, Ngondi J, Emerson PM (2009). The excess burden of trachomatous trichiasis in women: a systematic review and meta-analysis. Trans R Soc Trop Med Hyg.

[35] Courtright P, West SK (2004). Contribution of sex-linked biology and gender roles to disparities with trachoma. Emerg Infect Dis.

[36] West SK, Munoz B, Turner VM, Mmbaga BB, Taylor HR (1991). The epidemiology of trachoma in central Tanzania. Int J Epidemiol.

[37] Cahill KV, Foster JA, Black EH, Nesi FA, Calvano CJ, Gladstone GJ, Levine MR (2012). Trichiasis. Smith and Nesi’s ophthalmic plastic and reconstructive surgery.

[38] Brooks P, Rosenberg J, Weintraub R (2016). The global trachoma mapping project. Cases in Global Health delivery.

